# Supplementary material for: Exploring the Immediate Effects of COVID-19 Containment Policies on Crime: an Empirical Analysis of the Short-Term Aftermath in Los Angeles
Source: Am J Crim Justice. 2020 Oct 19;46(5):704–27. doi: 10.1007/s12103-020-09578-6 (PMC7571535; doi:10.1007/s12103-020-09578-6)
Supplement: Supplementary file 1 — (DOCX 33 kb) [file 12103_2020_9578_MOESM1_ESM.docx]

**SUPPLEMENTARY MATERIALS**

This document presents the complete statistical results from the Bayesian models performed per each crime category considered.

|  | *Up to March 16th* | | | |
| --- | --- | --- | --- | --- |
|  | Univariate | | With Cov. | |
|  | Avg. | Cum. | Avg. | Cum. |
| Actual | 26 | 317 | 26 | 317 |
| Prediction (S.D.) | 27 (2.2) | 327 (26.9) | 27 (2.2) | 327 (27.1) |
| 95% C.I. | [23,32] | [275,379] | [23,31] | [274, 374] |
| Absolute Effect (S.D.) | -0.79 (2.2) | -9.50 (26.9) | -0.41 (2.3) | -4.89 (27.1) |
| 95% C.I. | [-5.1, 3.5] | [-61.6, 42.2] | [-4.7, 3.6] | [-56.9, 43.1] |
| Relative Effect (S.D.) | -2.98% (8.2%) | -2.98% (8.2%) | -1.5% (8.4%) | -1.5% (8.4%) |
| 95% C.I. | [-19%, 13%] | [-19%, 13%] | [-18%, 13%] | [-18%, 13%] |
| Post. tail-area prob. p: | 0.36409 | | 0.48134 | |
| Post. prob. causal effect: | 64% | | 52% | |
|  | *Up to March 28th* | | | |
|  | Univariate | | With Cov. | |
|  | Avg. | Cum. | Avg. | Cum. |
| Actual | 24 | 604 | 24 | 604 |
| Prediction (S.D.) | 27 (1.8) | 675 (45.7) | 26 (1.5) | 644 (38.4) |
| 95% C.I. | [23, 30] | [585, 760] | [23, 29] | [568, 718] |
| Absolute Effect (S.D.) | -2.9 (1.8) | -71.3 (45.7) | -1.6 (1.5) | -40.4 (38.4) |
| 95% C.I. | [-6.2, 0.76] | [-155.6, 19.00] | [-4.6, 1.4] | [-114.2, 35.6] |
| Relative Effect (S.D.) | -11% (6.8%) | -11% (6.8%) | -6.3% (6%) | -6.3% (6%) |
| 95% C.I. | [-23%, 2.8%] | [-23%, 2.8%] | [-18%, 5.5%] | [-18%, 5.5%] |
| Post. tail-area prob. p: | 0.0625 | | 0.14801 | |
| Post. prob. causal effect: | 94% | | 85% | |

Table A 1. Causal Impact Analysis - Assaults With Deadly Weapons

|  | *Up to March 16th* | | | |
| --- | --- | --- | --- | --- |
|  | Univariate | | With Cov. | |
|  | Avg. | Cum. | Avg. | Cum. |
| Actual | 50 | 597 | 50 | 597 |
| Prediction (S.D.) | 50 (2.9) | 601 (34.4) | 49 (2.7) | 592 (32.5) |
| 95% C.I. | [44, 56] | [533, 668] | [44, 54] | [530, 652] |
| Absolute Effect (S.D.) | -0.3 (2.9) | -3.6 (34.4) | 0.39 (2.7) | 4.62 (32.5) |
| 95% C.I. | [-5.9, 5.3] | [-71.2, 64.1] | [-4.5, 5.6] | [-54.5, 66.7] |
| Relative Effect (S.D.) | -0.6% (5.7%) | -0.6% (5.7%) | 0.78% (5.5%) | 0.78% (5.5%) |
| 95% C.I. | [-12%, 11%] | [-12%, 11%] | [-9.2%, 11%] | [-9.2%, 11%] |
| Post. tail-area prob. p: | 0.41895 | | 0.40672 | |
| Post. prob. causal effect: | 58% | | 59% | |
|  | *Up to March 28th* | | | |
|  | Univariate | | With Cov. | |
|  | Avg. | Cum. | Avg. | Cum. |
| Actual | 45 | 1116 | 45 | 1116 |
| Prediction (S.D.) | 50 (2.5) | 1256 (63.1) | 48 (2) | 1207 (50) |
| 95% C.I. | [45, 55] | [1128, 1380] | [44, 52] | [1111, 1307] |
| Absolute Effect (S.D.) | -5.6 (2.5) | -139.7 (63.1) | -3.7 (2) | -91.3 (50) |
| 95% C.I. | [-11, -0.5] | [-264, -12.4] | [-7.6, 0.19] | [-191.1, 4.74] |
| Relative Effect (S.D.) | -11% (5%) | -11% (5%) | -7.6% (4.1%) | -7.6% (4.1%) |
| 95% C.I. | [-21%, -0.99%] | [-21%, -0.99%] | [-16%, 0.39%] | [-16%, 0.39%] |
| Post. tail-area prob. p: | 0.02486 | | 0.03534 | |
| Post. prob. causal effect: | 98% | | 96% | |

Table A 2. Causal Impact Analysis – Battery (Simple Assault)

|  | *Up to March 16th* | | | |
| --- | --- | --- | --- | --- |
|  | Univariate | | With Cov. | |
|  | Avg. | Cum. | Avg. | Cum. |
| Actual | 34 | 406 | 34 | 406 |
| Prediction (S.D.) | 34 (2.4) | 402 (29.2) | 34 (2.5) | 408 (29.6) |
| 95% C.I. | [29, 38] | [345, 461] | [30, 39] | [359, 465] |
| Absolute Effect (S.D.) | 0.3 (2.4) | 3.6 (29.2) | -0.2 (2.5) | -2.4 (29.6) |
| 95% C.I. | [-4.6, 5.1] | [-54.9, 60.8] | [-4.9, 3.9] | [-59.2, 46.6] |
| Relative Effect (S.D.) | 0.89% (7.2%) | 0.89% (7.2%) | -0.58% (7.2%) | -0.58% (7.2%) |
| 95% C.I. | [-14%, 15%] | [-14%, 15%] | [-14%, 11%] | [-14%, 11%] |
| Post. tail-area prob. p: | 0.44173 | | 0.49627 | |
| Post. prob. causal effect: | 56% | | 50% | |
|  | *Up to March 28th* | | | |
|  | Univariate | | With Cov. | |
|  | Avg. | Cum. | Avg. | Cum. |
| Actual | 33 | 816 | 33 | 816 |
| Prediction (S.D.) | 34 (1.9) | 858 (46.3) | 35 (1.8) | 881 (46.1) |
| 95% C.I. | [31, 38] | [769, 948] | [31, 39] | [787, 966] |
| Absolute Effect (S.D.) | -1.7 (1.9) | -41.6 (46.3) | -2.6 (1.8) | -64.5 (46.1) |
| 95% C.I. | [-5.3, 1.9] | [-131.7, 47.1] | [-6, 1.2] | [-150, 29.3] |
| Relative Effect (S.D.) | -4.8% (5.4%) | -4.8% (5.4%) | -7.3% (5.2%) | -7.3% (5.2%) |
| 95% C.I. | [-15%, 5.5%] | [-15%, 5.5%] | [-17%, 3.3%] | [-17%, 3.3%] |
| Post. tail-area prob. p: | 0.19832 | | 0.09328 | |
| Post. prob. causal effect: | 80% | | 91% | |

Table A 3. Causal Impact Analysis – Burglary

|  | *Up to March 16th* | | | |
| --- | --- | --- | --- | --- |
|  | Univariate | | With Cov. | |
|  | Avg. | Cum. | Avg. | Cum. |
| Actual | 38 | 454 | 38 | 454 |
| Prediction (S.D.) | 39 (2.3) | 473 (27.5) | 39 (2.1) | 466 (25.3) |
| 95% C.I. | [35, 44] | [424, 529] | [34, 43] | [414, 512] |
| Absolute Effect (S.D.) | -1.6 (2.3) | -18.8 (27.5) | -0.96 (2.1) | -11.52 (25.3) |
| 95% C.I. | [-6.3, 2.5] | [-75.4, 30.3] | [-4.9, 3.4] | [-58.4, 40.2] |
| Relative Effect (S.D.) | -4% (5.8%) | -4% (5.8%) | -2.5% (5.4%) | -2.5% (5.4%) |
| 95% C.I. | [-16%, 6.4%] | [-16%, 6.4%] | [-13%, 8.6%] | [-13%, 8.6%] |
| Post. tail-area prob. p: | 0.24535 | | 0.37313 | |
| Post. prob. causal effect: | 75% | | 63% | |
|  | *Up to March 28th* | | | |
|  | Univariate | | With Cov. | |
|  | Avg. | Cum. | Avg. | Cum. |
| Actual | 39 | 969 | 39 | 969 |
| Prediction (S.D.) | 39 (2.2) | 972 (56.0) | 38 (1.7) | 938 (41.7) |
| 95% C.I. | [34, 43] | [860, 1071] | [34, 41] | [855, 1021] |
| Absolute Effect (S.D.) | -0.11 (2.2) | -2.68 (56.0) | 1.2 (1.7) | 30.9 (41.7) |
| 95% C.I. | [-4.1, 4.4] | [-102.3, 109.3] | [-2.1, 4.5] | [-52.1, 113.7] |
| Relative Effect (S.D.) | -0.28% (5.8%) | -0.28% (5.8%) | 3.3% (4.4%) | 3.3% (4.4%) |
| 95% C.I. | [-11%, 11%] | [-11%, 11%] | [-5.6%, 12%] | [-5.6%, 12%] |
| Post. tail-area prob. p: | 0.48324 | | 0.22015 | |
| Post. prob. causal effect: | 52% | | 78% | |

Table A 4. Causal Impact Analysis - Intimate Partner Assault

|  | *Up to March 16th* | | | |
| --- | --- | --- | --- | --- |
|  | Univariate | | With Cov. | |
|  | Avg. | Cum. | Avg. | Cum. |
| Actual | 17 | 202 | 17 | 202 |
| Prediction (S.D.) | 22 (1.7) | 266 (20.5) | 22 (1.6) | 262 (19.5) |
| 95% C.I. | [19, 25] | [225, 304] | [19, 25] | [225, 302] |
| Absolute Effect (S.D.) | -5.3 (1.7) | -63.6 (20.5) | -5 (1.6) | -60 (19.5) |
| 95% C.I. | [-8.5, -1.9] | [-101.8, -22.6] | [-8.3, -1.9] | [-100.1, -22.8] |
| Relative Effect (S.D.) | -24% (7.7%) | -24% (7.7%) | -23% (7.5%) | -23% (7.5%) |
| 95% C.I. | [-38%, -8.5%] | [-38%, -8.5%] | [-38%, -8.7%] | [-38%, -8.7%] |
| Post. tail-area prob. p: | 0.00333 | | 0.00208 | |
| Post. prob. causal effect: | 99.67% | | 99.79% | |
|  | *Up to March 28th* | | | |
|  | Univariate | | With Cov. | |
|  | Avg. | Cum. | Avg. | Cum. |
| Actual | 18 | 439 | 18 | 439 |
| Prediction (S.D.) | 22 (1.3) | 553 (32.8) | 22 (1.2) | 545 (30.4) |
| 95% C.I. | [20, 25] | [490, 619] | [19, 24] | [486, 604] |
| Absolute Effect (S.D.) | -4.6 (1.3) | -114.2 (32.8) | -4.3 (1.2) | -106.3 (30.4) |
| 95% C.I. | [-7.2, -2.1] | [-180.5, -51.3] | [-6.6, -1.9] | [-165.4, -47.4] |
| Relative Effect (S.D.) | -21% (5.9%) | -21% (5.9%) | -19% (5.6%) | -19% (5.6%) |
| 95% C.I. | [-33%, -9.3%] | [-33%, -9.3%] | [-30%, -8.7%] | [-30%, -8.7%] |
| Post. tail-area prob. p: | 0.00298 | | 0.00218 | |
| Post. prob. causal effect: | 99.70% | | 99.78% | |

Table A 5. Causal Impact Analysis – Robbery

|  | *Up to March 16th* | | | |
| --- | --- | --- | --- | --- |
|  | Univariate | | With Cov. | |
|  | Avg. | Cum. | Avg. | Cum. |
| Actual | 16 | 189 | 16 | 189 |
| Prediction (S.D.) | 18 (1.5) | 220 (18.2) | 19 (1.5) | 223 (17.7) |
| 95% C.I. | [15, 21] | [184, 255] | [16, 21] | [188, 256] |
| Absolute Effect (S.D.) | -2.6 (1.5) | -31.1 (18.2) | -2.8 (1.5) | -34.1 (17.7) |
| 95% C.I. | [-5.5, 0.44] | [-66.3, 5.30] | [-5.6, 0.064] | [-67.4, 0.766] |
| Relative Effect (S.D.) | -14% (8.3%) | -14% (8.3%) | -15% (7.9%) | -15% (7.9%) |
| 95% C.I. | [-30%, 2.4%] | [-30%, 2.4%] | [-30%, 0.34%] | [-30%, 0.34%] |
| Post. tail-area prob. p: | 0.04353 | | 0.00208 | |
| Post. prob. causal effect: | 95.64% | | 99.79% | |
|  | *Up to March 28th* | | | |
|  | Univariate | | With Cov. | |
|  | Avg. | Cum. | Avg. | Cum. |
| Actual | 13 | 320 | 13 | 320 |
| Prediction (S.D.) | 18 (1) | 462 (26) | 19 (1) | 471 (26) |
| 95% C.I. | [16, 21] | [412, 513] | [17, 21] | [421, 521] |
| Absolute Effect (S.D.) | -5.7 (1) | -142.0 (26) | -6.1 (1) | -151.5 (26) |
| 95% C.I. | [-7.7, -3.7] | [-193.4, -92.0] | [-8, -4.1] | [-201, -101.3] |
| Relative Effect (S.D.) | -31% (5.6%) | -31% (5.6%) | -32% (5.5%) | -32% (5.5%) |
| 95% C.I. | [-42%, -20%] | [-42%, -20%] | [-43%, -21%] | [-43%, -21%] |
| Post. tail-area prob. p: | 0.00348 | | 0.001 | |
| Post. prob. causal effect: | 99.65% | | 99.90% | |

Table A 6. Causal Impact Analysis - Shoplifting

|  | *Up to March 16th* | | | |
| --- | --- | --- | --- | --- |
|  | Univariate | | With Cov. | |
|  | Avg. | Cum. | Avg. | Cum. |
| Actual | 55 | 662 | 55 | 662 |
| Prediction (S.D.) | 61 (2.9) | 728 (35.3) | 61 (2.8) | 732 (33.2) |
| 95% C.I. | [55, 66] | [658, 797] | [56, 67] | [670, 801] |
| Absolute Effect (S.D.) | -5.5 (2.9) | -66.1 (35.3) | -5.8 (2.8) | -70.0 (33.2) |
| 95% C.I. | [-11, 0.34] | [-135, 4.13] | [-12, -0.64] | [-139, -7.67] |
| Relative Effect (S.D.) | -9.1% (4.8%) | -9.1% (4.8%) | -9.6% (4.5%) | -9.6% (4.5%) |
| 95% C.I. | [-19%, 0.57%] | [-19%, 0.57%] | [-19%, -1%] | [-19%, -1%] |
| Post. tail-area prob. p: | 0.0333 | | 0.01663 | |
| Post. prob. causal effect: | 96.67% | | 98.33% | |
|  | *Up to March 28th* | | | |
|  | Univariate | | With Cov. | |
|  | Avg. | Cum. | Avg. | Cum. |
| Actual | 47 | 1175 | 47 | 1175 |
| Prediction (S.D.) | 62 (2.2) | 1548 (56.2) | 62 (2.1) | 1557 (53.4) |
| 95% C.I. | [58, 66] | [1444, 1651] | [58, 66] | [1453, 1658] |
| Absolute Effect (S.D.) | -15 (2.2) | -373 (56.2) | -15 (2.1) | -382 (53.4) |
| 95% C.I. | [-19, -11] | [-476, -269] | [-19, -11] | [-483, -278] |
| Relative Effect (S.D.) | -24% (3.6%) | -24% (3.6%) | -25% (3.4%) | -25% (3.4%) |
| 95% C.I. | [-31%, -17%] | [-31%, -17%] | [-31%, -18%] | [-31%, -18%] |
| Post. tail-area prob. p: | 0.0035 | | 0.00109 | |
| Post. prob. causal effect: | 99.65% | | 99.89% | |

Table A 7. Causal Impact Analysis – Theft

|  | *Up to March 16th* | | | |
| --- | --- | --- | --- | --- |
|  | Univariate | | With Cov. | |
|  | Avg. | Cum. | Avg. | Cum. |
| Actual | 45 | 536 | 45 | 536 |
| Prediction (S.D.) | 44 (2.4) | 530 (28.6) | 45 (2.4) | 536 (28.8) |
| 95% C.I. | [40, 49] | [477, 586] | [40, 49] | [483, 591] |
| Absolute Effect (S.D.) | 0.46 (2.4) | 5.55 (28.6) | 0.029 (2.4) | 0.343 (28.8) |
| 95% C.I. | [-4.2, 4.9] | [-49.8, 58.8] | [-4.6, 4.4] | [-55.1, 52.8] |
| Relative Effect (S.D.) | 1% (5.4%) | 1% (5.4%) | 0.064% (5.4%) | 0.064% (5.4%) |
| 95% C.I. | [-9.4%, 11%] | [-9.4%, 11%] | [-10%, 9.9%] | [-10%, 9.9%] |
| Post. tail-area prob. p: | 0.41646 | | 0.48507 | |
| Post. prob. causal effect: | 58% | | 51% | |
|  | *Up to March 28th* | | | |
|  | Univariate | | With Cov. | |
|  | Avg. | Cum. | Avg. | Cum. |
| Actual | 46 | 1140 | 46 | 1140 |
| Prediction (S.D.) | 45 (2) | 1123 (49) | 46 (1.7) | 1141 (43.3) |
| 95% C.I. | [41, 49] | [1032, 1214] | [42, 49] | [1054, 1225] |
| Absolute Effect (S.D.) | 0.67 (2) | 16.84 (49) | -0.055 (1.7) | -1.369 (43.3) |
| 95% C.I. | [-2.9, 4.3] | [-73.6, 107.8] | [-3.4, 3.4] | [-85.0, 86.2] |
| Relative Effect (S.D.) | 1.5% (4.4%) | 1.5% (4.4%) | -0.12% (3.8%) | -0.12% (3.8%) |
| 95% C.I. | [-6.5%, 9.6%] | [-6.5%, 9.6%] | [-7.4%, 7.5%] | [-7.4%, 7.5%] |
| Post. tail-area prob. p: | 0.0035 | | 0.00109 | |
| Post. prob. causal effect: | 99.65% | | 99.89% | |

Table A 8. Causal Impact Analysis - Stolen Vehicles

|  | *Up to March 16th* | | | |
| --- | --- | --- | --- | --- |
|  | Univariate | | With Cov. | |
|  | Avg. | Cum. | Avg. | Cum. |
| Actual | 0.62 | 8.00 | 0.62 | 8.00 |
| Prediction (S.D.) | 0.72 (0.26) | 9.38 (3.36) | 0.69 (0.25) | 8.94 (3.28) |
| 95% C.I. | [0.21, 1.3] | [2.69, 16.3] | [0.21, 1.2] | [2.73, 15.5] |
| Absolute Effect (S.D.) | -0.11 (0.26) | -1.38 (3.36) | -0.072 (0.25) | -0.935 (3.28) |
| 95% C.I. | [-0.64, 0.41] | [-8.28, 5.31] | [-0.58, 0.41] | [-7.48, 5.27] |
| Relative Effect (S.D.) | -15% (36%) | -15% (36%) | -10% (37%) | -10% (37%) |
| 95% C.I. | [-88%, 57%] | [-88%, 57%] | [-84%, 59%] | [-84%, 59%] |
| Post. tail-area prob. p: | 0.326 | | 0.37437 | |
| Post. prob. causal effect: | 67% | | 63% | |
|  | *Up to March 28th* | | | |
|  | Univariate | | With Cov. | |
|  | Avg. | Cum. | Avg. | Cum. |
| Actual | 0.52 | 13.00 | 0.52 | 13.00 |
| Prediction (S.D.) | 0.72 (0.19) | 18.11 (4.83) | 0.69 (0.19) | 17.14 (4.84) |
| 95% C.I. | [0.34, 1.1] | [8.39, 27.3] | [0.31, 1] | [7.71, 26] |
| Absolute Effect (S.D.) | -0.2 (0.19) | -5.1 (4.83) | -0.17 (0.19) | -4.14 (4.84) |
| 95% C.I. | [-0.57, 0.18] | [-14.34, 4.61] | [-0.52, 0.21] | [-13.04, 5.29] |
| Relative Effect (S.D.) | -28% (27%) | -28% (27%) | -24% (28%) | -24% (28%) |
| 95% C.I. | [-79%, 25%] | [-79%, 25%] | [-76%, 31%] | [-76%, 31%] |
| Post. tail-area prob. p: | 0.13814 | | 0.2012 | |
| Post. prob. causal effect: | 86% | | 80% | |

Table A 9. Causal Impact Analysis – Homicides

|  | *Up to March 16th* | | | |
| --- | --- | --- | --- | --- |
|  | Univariate | | With Cov. | |
|  | Avg. | Cum. | Avg. | Cum. |
| Actual | 558 | 6700 | 558 | 6700 |
| Prediction (S.D.) | 592 (14) | 7098 (165) | 590 (13) | 7086 (152) |
| 95% C.I. | [567, 620] | [6805, 7441] | [564, 615] | [6772, 7376] |
| Absolute Effect (S.D.) | -33 (14) | -398 (165) | -32 (13) | -386 (152) |
| 95% C.I. | [-62, -8.8] | [-741, -105.1] | [-56, -6] | [-676, -72] |
| Relative Effect (S.D.) | -5.6% (2.3%) | -5.6% (2.3%) | -5.4% (2.1%) | -5.4% (2.1%) |
| 95% C.I. | [-10%, -1.5%] | [-10%, -1.5%] | [-9.5%, -1%] | [-9.5%, -1%] |
| Post. tail-area prob. p: | 0.00555 | | 0.01493 | |
| Post. prob. causal effect: | 99.44% | | 98.51% | |
|  | *Up to March 28th* | | | |
|  | Univariate | | With Cov. | |
|  | Avg. | Cum. | Avg. | Cum. |
| Actual | 513 | 12829 | 513 | 12829 |
| Prediction (S.D.) | 600 (12) | 14893 (49) | 596 (9) | 14899 (224) |
| 95% C.I. | [580, 624] | [14490, 15600] | [580, 614] | [14500, 15341] |
| Absolute Effect (S.D.) | -87 (12) | -2182 (300) | -83 (9) | -2070 (224) |
| 95% C.I. | [-111, -66] | [-2771, -1661] | [-100, -67] | [-2512, -1671] |
| Relative Effect (S.D.) | -15% (2%) | -15% (2%) | -14% (1.5%) | -14% (1.5%) |
| 95% C.I. | [-18%, -11%] | [-18%, -11%] | [-17%, -11%] | [-17%, -11%] |
| Post. tail-area prob. p: | 0.00279 | | 0.00208 | |
| Post. prob. causal effect: | 99.72% | | 99.79% | |

Table A 10. Causal Impact Analysis - Overall Crimes
